# p62/SQSTM1 cooperates with Parkin for perinuclear clustering of depolarized mitochondria

**DOI:** 10.1111/j.1365-2443.2010.01426.x

**Published:** 2010-08

**Authors:** Kei Okatsu, Keiko Saisho, Midori Shimanuki, Kazuto Nakada, Hiroshi Shitara, Yu-shin Sou, Mayumi Kimura, Shigeto Sato, Nobutaka Hattori, Masaaki Komatsu, Keiji Tanaka, Noriyuki Matsuda

**Affiliations:** 1 Laboratory of Protein Metabolism, Tokyo Metropolitan Institute of Medical ScienceSetagaya-ku, Tokyo 156-8506, Japan; 2 Graduate School of Medical and Dental Sciences, Niigata UniversityAsahimachi, Niigata 951-8510, Japan; 3 Laboratory for Transgenic Technology, Tokyo Metropolitan Institute of Medical ScienceSetagaya-ku, Tokyo 156-8506, Japan; 4 Graduate School of Life and Environmental Sciences, University of TsukubaTennodai, Tsukuba, Ibaraki 305-8572, Japan; 5 Department of Neurology, Juntendo University School of MedicineBunkyo-ku, Tokyo 113-8421, Japan; 6 PRESTO, Japan Science and Technology CorporationKawaguchi 332-0012, Japan

## Abstract

*PINK1* and *Parkin* were first identified as the causal genes responsible for familial forms of early-onset Parkinson’s disease (PD), a prevalent neurodegenerative disorder. *PINK1* encodes a mitochondrial serine/threonine protein kinase, whereas Parkin encodes an ubiquitin-protein ligase. PINK1 and Parkin cooperate to maintain mitochondrial integrity; however, the detailed molecular mechanism of how Parkin-catalyzed ubiquitylation results in mitochondrial integrity remains an enigma. In this study, we show that Parkin-catalyzed K63-linked polyubiquitylation of depolarized mitochondria resulted in ubiquitylated mitochondria being transported along microtubules to cluster in the perinuclear region, which was interfered by pathogenic mutations of Parkin. In addition, p62/SQSTM1 (hereafter referred to as p62) was recruited to depolarized mitochondria after Parkin-directed ubiquitylation. Intriguingly, deletion of p62 in mouse embryonic fibroblasts resulted in a gross loss of mitochondrial perinuclear clustering but did not hinder mitochondrial degradation. Thus, p62 is required for ubiquitylation-dependent clustering of damaged mitochondria, which resembles p62-mediated ‘aggresome’ formation of misfolded/unfolded proteins after ubiquitylation.

## Introduction

Our understanding of the pathogenesis of two autosomal recessive familial Parkinson’s diseases (PDs) has been greatly developed and continues to evolve. One familial form of the PD gene causing autosomal recessive juvenile Parkinsonism (AR-JP) is *Parkin* (also known as *PARK2*), which encodes an E3 ubiquitin ligase, a substrate recognition member of the ubiquitylation pathway (Kitada *et al.* 1998; Shimura *et al.* 2000). The other early-onset PD gene encodes a mitochondria-targeted serine-threonine kinase termed PTEN induced putative kinase 1 (PINK1) (Valente *et al.* 2004). Judging from their molecular functions, it is clear that Parkin-mediated ubiquitylation and phosphorylation by PINK1 are key events in disease pathogenesis, but the details are still largely unknown. Various clinical symptoms in patients caused by *Parkin* and *PINK1* dysfunctions resemble each other. In addition, genetic studies using *Drosophila* showed that PINK1 and Parkin function in the same mitochondrial homeostasis pathway, because individual loss of either of the two genes leads to abnormal mitochondrial morphology and integrity. Moreover, PINK1 functions upstream of Parkin, because forced overexpression of Parkin rescues loss-of-function phenotypes of PINK1 but not *vice versa* (Clark *et al.* 2006; Park *et al.* 2006; Yang *et al.* 2006).

Interestingly, PINK1 is rapidly and constitutively degraded under steady-state conditions in a normal (healthy) mitochondria, and a loss in mitochondrial membrane potential stabilizes PINK1 on damaged mitochondria by likely inhibiting an as-yet-unknown processing protease that catalyzes conversion of the mature PINK1 (approximately 60 kDa) to an intermediate short form (approximately 50 kDa), which is further exclusively degraded by the proteasome pathway (Matsuda *et al.* 2010; Narendra *et al.* 2010). PINK1 then recruits Parkin from the cytoplasm to depolarized mitochondria that then undergo Parkin-catalyzed ubiquitylation, meaning that PINK1 and Parkin cooperate to ubiquitylate mitochondria with low membrane potentials (Narendra *et al.* 2008, 2010; Geisler *et al.* 2010; Matsuda *et al.* 2010; Vives-Bauza *et al.* 2010).

Mitochondrial homeostasis plays a pivotal role in the maintenance of normal healthy cells, in particular nondividing cells such as neurons. To maintain the integrity of mitochondria, the selective elimination of impaired mitochondria caused by various endogenous and exogenous stresses, such as unnecessary generation of reactive oxygen species (ROS) and mtDNA mutations, is critical, with mitochondria-dedicated selective autophagy (termed mitophagy) considered to be essential for this clearance pathway (Narendra *et al.* 2008; Twig *et al.* 2008). Thus, it becomes clear that PINK1 and Parkin function in mitochondrial quality control; however, the detailed molecular mechanism of how Parkin-catalyzed ubiquitylation results in mitochondrial integrity remains an open question. Here, we show that Parkin leads to juxtanuclear clustering of depolarized mitochondria reminiscent of ‘aggresome’ formation and that the ubiquitin-interacting protein, p62/SQSTM1/sequestosome-1 (referred to hereafter as p62), is involved in this process.

## Results

### Parkin-catalyzed K63-linked polyubiquitylation promotes mitochondrial clustering

**Figure 1:**
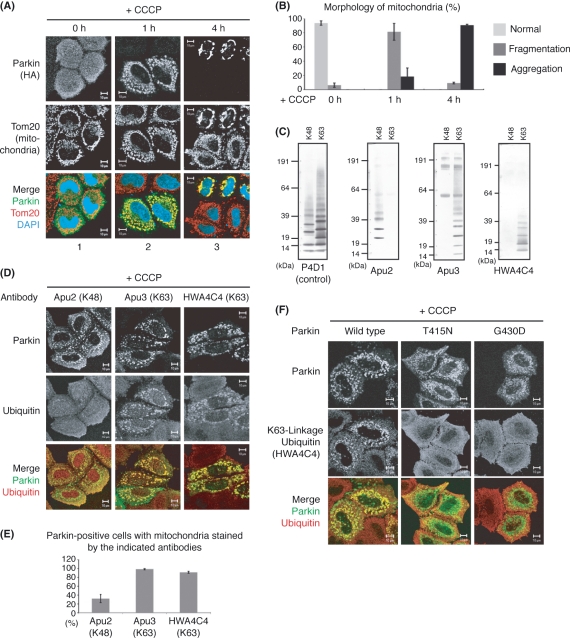
K63-linkage-specific anti-ubiquitin antibody can detect mitochondrial ubiquitylation catalyzed by Parkin. (A) HeLa cells expressing HA-Parkin were treated with CCCP for the indicated times, then immunostained with anti-HA or anti-Tom20 antibodies. Mitochondria clustered to the juxtanuclear region (*t* = 4 h) only in Parkin-expressing cells. (B) Morphology of mitochondria in Parkin-expressing HeLa cells was analyzed in more than 100 cells in each time course. Bars represent the mean ± SD values of at least three experiments. (C) Linkage-specificity of anti-ubiquitin antibodies. K48- or K63-linked ubiquitin chain was blotted with Apu2, Apu3 or HWA4C4 antibody. P4D1 recognizes any type of ubiquitin and was used as a positive control. (D) HeLa cells expressing HA-Parkin were treated with CCCP and subjected to immunocytochemistry with the indicated linkage-specific anti-ubiquitin antibodies. (E) Parkin-expressing HeLa cells were stained with the indicated antibodies, and cells with double-stained mitochondria by Parkin and linkage-specific Ub were counted in more than 100 cells per antibody. Bars represent the mean ± SD values of at least three experiments. (F) K63-linked ubiquitylation on mitochondria depends on the E3 activity of Parkin. K63-linked ubiquitylation signal disappeared when the catalytically dead T415N and G430D mutations were introduced into Parkin. Scale bars represent 10 μm in (A), (D) and (F).

As reported by us and other groups, a loss of mitochondrial membrane potential triggers stabilization of mitochondrial PINK1. This drastic accumulation of PINK1 then serves to recruit Parkin, which ubiquitylates the outer membrane protein(s) of mitochondria with no membrane potential with the ubiquitylated mitochondria destined for final degradation in part via mitophagy (Narendra *et al.* 2008, 2010; Geisler *et al.* 2010; Matsuda *et al.* 2010; Vives-Bauza *et al.* 2010; Ziviani *et al.* 2010). To dissect this process in more detail, we analyzed the mode of ubiquitylation and time course of mitochondria degradation in depth. In mitochondria fragmented with the mitochondrial uncoupler, carbonyl cyanide m-chlorophenylhydrazone (CCCP), Parkin quickly moved to depolarized mitochondria and ubiquitylated them (*t* = 1 h). Interestingly, mitochondria later concentrated to the perinuclear region at *t* > 4 h (Fig. 1A,B) and finally degraded as reported (Narendra *et al.* 2008) (not shown). This perinuclear transport and clustering of mitochondria are Parkin dependent, because this phenomenon was only observed in Parkin-expressing HeLa cells that lack an endogenous Parkin gene (Denison *et al.* 2003) (Fig. 1A, panel 3).

Because the fate of ubiquitylated proteins is determined by which lysine(s) within ubiquitin is covalently linked via an isopeptide bond to the C terminus of an adjacent ubiquitin (Weissman 2001), we next tried to determine the linkage mode of ubiquitylation catalyzed by Parkin. Parkin has the potential to catalyze several types of ubiquitylation: multiple monoubiquitylation, Lys-48-linked polyubiquitylation and Lys-63-linked polyubiquitylation (Lim 2007; Matsuda & Tanaka 2010). Recently, Springer *et al.* reported that Parkin catalyzes poly-ubiquitin chain linked through K27 and K63 on depolarized mitochondria (Geisler *et al.* 2010). However, in their work, various ubiquitin mutants such as 63K-only or K63R, whose lysine residue is replaced by an arginine residue, were over-expressed to determine the linkage of Parkin-catalyzed ubiquitylation. However, overproduction of mutant ubiquitin can change the ubiquitylation linkage, and thus careful interpretation of observed results is required (Pickart & Raasi 2005; Saeki *et al.* 2009). To address this issue, we used linkage-specific anti-ubiquitin antibodies Apu2, Apu3 and HWA4C4 (Newton *et al.* 2008; Wang *et al.* 2008) that can determine the linkage without overproduction of mutant ubiquitin. Immunoblotting against K48- and K63-linked polyubiquitin chains, whose linkage was directly confirmed by MALDI-TOF mass spectrometry (data not shown), showed the high specificity of the antibodies (Fig. 1C). In immunocytochemistry experiments, the Apu3 and HWA4C4 antibodies, which exclusively react with ubiquitin chain linked at K63, clearly stained Parkin-localized mitochondria, whereas Apu2, which specifically recognizes K48-linkage polyubiquitylation, did not stain Parkin-localized mitochondria as well (Fig. 1D,E). We confirmed that the signals generated by Apu3 and HWA4C4 disappeared with E3-inactivated mutations of Parkin (Fig. 1F and not shown). These results suggest that Parkin ubiquitylates the damaged mitochondria mainly via K63-linked polyubiquitylation and are consistent, in part, with the recent report by Geisler *et al.* (2010).

### Mitochondrial clustering is microtubule dependent

**Figure 2:**
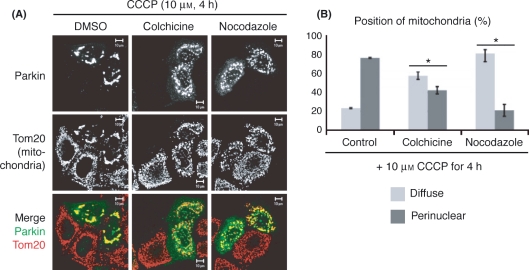
Mitochondrial clustering catalyzed by Parkin is microtubule dependent. (A) Microtubule polymerization inhibitors hampered the perinuclear clustering of mitochondria. HeLa cells expressing HA-Parkin were treated with CCCP plus DMSO (control), nocodazole or colchicine, then immunostained with anti-Parkin or anti-Tom20 antibodies. Scale bars represent 10 μm. Treatment with both nocodazole and colchicine caused the depolarized mitochondria to scatter throughout the cells. (B) The morphology of mitochondria was analyzed in more than 100 cells per each condition. Bars represent the mean ± SD values of at least three experiments. Asterisk, *P* < 0.01 (Welch’s *t*-test).

Within the cell, many organelles including mitochondria are transported along microtubules, which function as intracellular railways (Hirokawa 1998). To examine whether microtubules are involved in Parkin-dependent mitochondrial-clustering, we carried out pharmacologic experiments. Parkin-expressing HeLa cells were treated with nocodazole and colchicine, both of which inhibit microtubule polymerization. After treatment with nocodazole (10 μg/mL) or colchicine (50 μg/mL), the perinuclear mitochondria clusters dissipated (Fig. 2A). These data and statistical analyses (Fig. 2B) showed that mitochondrial morphology was altered from a large cluster to small aggregates scattered throughout the cells. In contrast, treatment with latrunculin A/B, which prevents actin polymerization, had no effect on the mitochondrial perinuclear clustering (not shown). These results suggest that the transport of depolarized mitochondria to the perinuclear region is microtubule dependent and are in agreement with a recent report by Przedborski’s group (Vives-Bauza *et al.* 2010).

### Various pathogenic mutations of Parkin impede mitochondrial clustering

**Figure 3:**
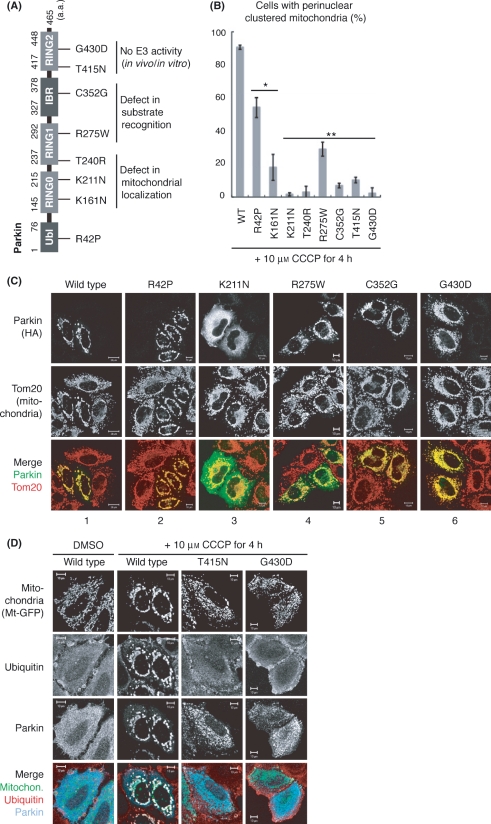
Various pathogenic mutations of Parkin impede mitochondrial clustering. (A) Schematic diagram of disease-relevant mutants of Parkin used in this study. IBR, in between RING; Ubl, ubiquitin like. (B) HeLa cells expressing HA-Parkin with various pathogenic mutations were treated with CCCP followed by immunocytochemistry. Mitochondrial clustering was analyzed in more than 100 cells per mutation. Bars represent the mean ± SD values of at least three experiments. Asterisk, *P* < 0.01; two asterisks, *P* < 0.001 (Welch’s *t*-test). (C) Immunocytochemistry indicative of a typical example for each mutation is shown. Scale bars represent 20 μm in wild type and 10 μm in the other mutations. (D) Triple-staining using mitochondria-targeting GFP (Mt-GFP), anti-ubiquitin and anti-Parkin antibodies confirmed that mitochondria in T415N and G430D mutants did not undergo ubiquitylation or clustering.

We next examined whether pathogenic mutations of Parkin affect the perinuclear clustering of mitochondria. HA-Parkin mutants harboring one of eight pathogenic mutations (R42P, K161N, T240R, R275W, C352G, T415N and G430D; Fig. 3A) were serially introduced into HeLa cells, followed by CCCP treatment for 4 h and the mitochondrial morphology analyzed in more than 100 cells per mutation (Fig. 3B). The K161N, K211N and T240R mutations, which severely compromised the mitochondrial localization of Parkin (Geisler *et al.* 2010; Matsuda *et al.* 2010; Narendra *et al.* 2010), inhibited the mitochondrial clustering. In these cells and in particular the K211N cells, Parkin was diffusely localized throughout the cytosol and did not affect mitochondrial clustering (Fig. 3C, panel 3). In the R275W and C352G mutations, Parkin associated with depolarized mitochondria, but mitochondrial ubiquitylation and juxtanuclear clustering were severely inhibited (Fig. 3C and not shown). Because ubiquitin-ligase (E3) activity against a pseudo-substrate (i.e., the MBP moiety of the MBP-fused Parkin *in vitro* and the GFP moiety of the GFP-Parkin in cell) was intact in these Parkin mutations (Hampe *et al.* 2006; Matsuda *et al.* 2006, 2010), it is possible that the R275W and C352G mutations might affect the recognition of intrinsic substrates. Furthermore, mutations in the RING2 catalytic domain (T415N and G430D), which abolished the E3 activity of Parkin *in vitro* (Hampe *et al.* 2006; Matsuda *et al.* 2006), impeded the mitochondrial clustering (Fig. 3B). In these cells, even when mutant Parkin localized on damaged mitochondria, mitochondria were still dissipated throughout the cells (Fig. 3C, panel 6). Triple staining for mitochondria, ubiquitin and Parkin further confirmed that the T415N and G430D mutants did not promote mitochondrial ubiquitylation or mitochondrial clustering (Fig. 3D). These results suggest that ubiquitylation catalyzed by Parkin is required for mitochondrial clustering.

### p62/SQSTM1 is not essential for mitochondrial degradation

What then links the ubiquitylation of depolarized mitochondria to their perinuclear clustering? The important clue was obtained from our study focusing on the role of p62 in Parkin-mediated mitochondrial degradation. p62 can interact with both ubiquitin and the autophagic machinery LC-3 (Bjorkoy *et al.* 2005; Komatsu *et al.* 2007; Pankiv *et al.* 2007; Ichimura *et al.* 2008; Noda *et al.* 2008; Shvets *et al.* 2008) and thus p62 is a plausible factor that links Parkin-catalyzed ubiquitylation with mitochondrial degradation via autophagy.

**Figure 4:**
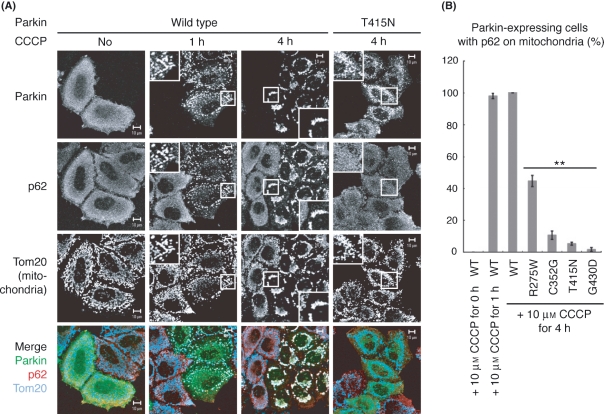
E3 activity of Parkin recruits endogenous p62 to depolarized mitochondria. (A) HeLa cells expressing wild type or E3-incompetent Parkin were treated with CCCP for 0, 1 or 4 h, then immunostained with the indicated antibodies. Note that endogenous p62 was recruited to mitochondria only in wild-type Parkin-expressing cells. All scale bars represent 10 μm and higher magnification views of the boxed areas are shown in the insets. (B) Recruitment of p62 onto mitochondria was analyzed in more than 100 cells in each experimental condition. Bars represent the mean ± SD values of at least three experiments. Two asterisks, *P* < 0.001 (Welch’s *t*-test).

Under steady-state conditions in HeLa cells, endogenous p62 was mainly localized throughout the cytosol irrespective of the presence or absence of Parkin, whereas p62 was rapidly recruited to the mitochondria only in Parkin-expressing cells after CCCP treatment for 1 h (Fig. 4A), as also reported by Springer *et al.* recently (Geisler *et al.* 2010). Even after further treatment with CCCP for 4 h, p62 remained localized on perinuclear clustered-mitochondria. Staining with single antibodies alone indicated that the merged data described earlier were not derived from channel crosstalk (Fig. S1 in Supporting Information). Mitochondrial localization of p62 was only observed in Parkin-expressing cells and disappeared when Parkin mutants deficient in substrate recognition or E3 activity (R275W, C352G, T415N and G430D; Fig. 3A) were introduced (Fig. 4), suggesting that ubiquitylation-catalyzed Parkin is required for the recruitment of p62 onto depolarized mitochondria.

**Figure 5:**
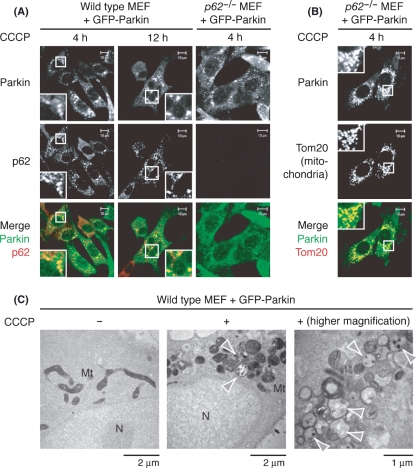
Damaged mitochondria clustered to the juxtanuclear region in mouse embryonic fibroblasts (MEFs). (A) Endogenous p62 was recruited to mitochondria in MEFs. Exogenous Parkin and endogenous p62 were stained in wild-type or *p62*^*−/−*^ MEFs. (B) Parkin was recruited to the mitochondria after CCCP treatment in *p62*^*−/−*^ MEFs. In A and B, higher magnification views of the boxed areas are shown in the insets. (C) Depolarized mitochondria in the perinuclear region were not fused to each other. Electron microscopic analysis showed that juxtanuclear-clustered mitochondria are aggregated like a bunch of grapes. Arrowheads indicate malformed mitochondria with disintegrated cristae. Scale bars for each figure are shown below.

To examine whether p62 is involved in mitochondrial degradation, we set up an experimental system using mouse embryonic fibroblasts (MEFs). Because endogenous Parkin is undetectable in MEFs, HA- or GFP-Parkin was introduced into control (*p62*^+/+^) and *p62* KO (*p62*^−/−^) MEFs (Komatsu *et al.* 2007) by retro-viral transfection (Kitamura *et al.* 2003). Because MEFs are less sensitive than HeLa cells to CCCP, higher concentrations of CCCP (30 μm) and longer incubation are required to observe the phenotype. Parkin was selectively recruited to the mitochondria after CCCP treatment in both *p62*^+/+^ and *p62*^−/−^ MEFs at *t* = 4 h (Fig. 5B and data not shown). In wild-type MEFs, p62 was again selectively recruited to the mitochondria after CCCP treatment at *t* = 4 h, although the transport efficiency was lower than that of HeLa cells (data not shown), and depolarized mitochondria were later clustered in the perinuclear region similar to HeLa cells at *t* > 12 h (Fig. 5A). We next carried out cytochrome c oxidase (COX) electronmicrographs that detect COX activity with mitochondria stained black (Fig. 5C, left panel) (Seligman *et al.* 1968). Electron microscopic analysis showed that juxtanuclear-clustered mitochondria aggregated in grape-like clusters and not fused to each other to make single large mitochondrion (Fig. 5C). Some mitochondria were malformed with disintegration of cristae structures indicated by black staining (Fig. 5C, arrowheads).

**Figure 6:**
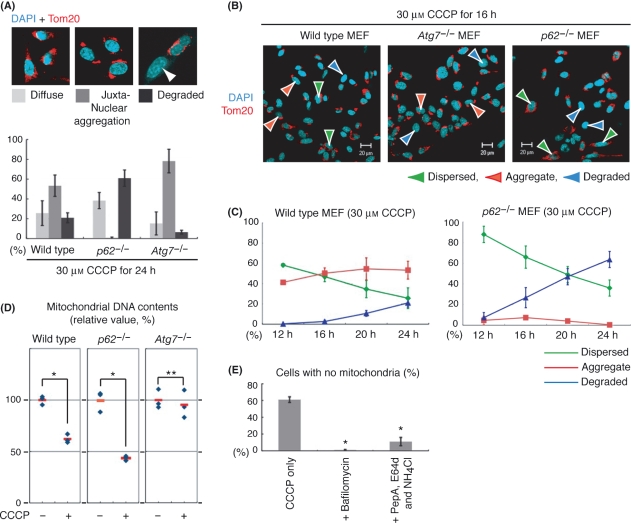
Juxtanuclear clustering rather than degradation of mitochondria is considerably inhibited in *p62*^*−/−*^ mouse embryonic fibroblasts (MEFs). (A) Top panel: example figures indicative of diffused (light gray in the bar graph), aggregated (gray) or degraded mitochondria (black) are shown. Bottom panel: wild type, *p62*^*−/−*^ and *Atg7* ^*−/−*^ MEFs expressing HA-Parkin were treated with CCCP for 24 h, and mitochondrial morphology was analyzed in >100 cells per mutation. Error bars represent the mean ± SD values of at least three experiments. (B) Immunocytochemistry of wild type, *p62*^*−/−*^ and *Atg7* ^*−/−*^ MEFs expressing HA-Parkin after CCCP treatment for 16 h. Green, red and blue arrowheads indicate cells with dispersed, aggregated and degraded mitochondria, respectively. (C) Time course experiments showing the morphologic change of mitochondria. Wild-type or *p62*^*−/−*^ MEFs expressing HA-Parkin were treated with CCCP for the indicated times, immunostained with anti-HA and anti-Tom20 antibodies, and then the mitochondrial morphology was analyzed in more than 100 cells per condition. Green, red and blue lines indicate cells with dispersed, aggregated and degraded mitochondria, respectively. Error bars represent the mean ± SD values of at least three experiments. (D) Measurement of mtDNA contents in wild type, *Atg7* ^*−/−*^ and *p62*^*−/−*^ MEFs after CCCP treatment. The graph represents the results from three independent experiments. Contents of mtDNA without CCCP treatment were defined as 100% in each cell. Asterisk shows significant difference from control (*P* < 0.01; Welch’s *t*-test), whereas two asterisks show no significance (*P* > 0.01). (E) Depolarized mitochondria are degraded by the autophagic and lysosomal pathway in *p62*^*−/−*^ MEFs. *p62*^*−/−*^ MEFs were treated with CCCP plus DMSO (control), bafilomycin A_1_ or a protease inhibitor cocktail (pepstatin A, E64d and NH_4_Cl), then the morphology of mitochondria was analyzed in more than 100 cells per each condition. Bars represent the mean ± SD values of at least three experiments. Asterisk, *P* < 0.001 (Welch’s *t*-test).

We then studied mitochondrial clearance in *p62*-ablated MEFs to examine whether p62 recognizes Parkin-catalyzed ubiquitylation on mitochondria and directs mitochondria to autophagosomes via binding to LC3. When *Atg7* (an essential gene for autophagy) KO MEFs (Komatsu *et al.* 2005) were used as a positive control, the clearance of depolarized mitochondria was considerably impeded after CCCP treatment for 24 h [Fig. 6A and (Matsuda *et al.* 2010)]. We then examined mitochondrial degradation in *p62*^−/−^ MEFs. Contrary to our expectations, immunocytochemistry experiments at 12, 16, 20 and 24 h showed that *p62* deletion did not inhibit but rather accelerated Parkin-dependent mitochondrial degradation (Fig. 6A–C). Immunocytochemistry using an anti-p62 antibody confirmed that endogenous *p62* was indeed lost in *p62*^−/−^ MEFs (Fig. 5A, right panel).

To quantitatively confirm the aforemetioned results, we examined the mtDNA copy number in wild-type, *Atg7*^−/−^ and *p62*^−/−^ MEFs after CCCP treatment. When we measured the abundance of mitochondrial 12s ribosomal RNA gene (encoded in mitochondrial DNA) normalized to the glyceraldehyde-3-phosphate dehydrogenase (GAPDH) gene (encoded in nuclear DNA) in wild-type MEFs, we found that the quantity of 12s ribosomal RNA gene was decreased to 62 ± 4% after CCCP treatment, whereas the *Atg7* knockout compensated for the decease by restoring the level to 95 ± 13%, suggesting that the reduction in mitochondrial DNA reflects Parkin-dependent mitophagy. Furthermore, CCCP treatment of *p62* KO MEFs promoted the degradation of mitochondrial DNA (43 ± 2%) comparable to that of wild-type MEFs (Fig. 6D), again implying that mitochondrial degradation occurs in *p62*^*−/−*^ MEFs. Moreover, treatment with bafilomycin A_1_ (an inhibitor of vacuolar-type H^+^-ATPase that prevents autophagosome-lysosome fusion and/or intralysosomal degradation) or a cocktail of lysosomal inhibitors (E64d, pepstatin A and ammonium chloride) (Mizushima *et al.* 2010) inhibited the mitochondrial degradation of *p62*^*−/−*^ MEFs by immunocytochemistry (Fig. 6E), showing that depolarized mitochondria were indeed degraded by the autophagic and lysosomal pathway in *p62*^*−/−*^ MEFs. Although these results do not rule out completely the involvement of p62 in Parkin-mediated mitophagy, a role for p62 in Parkin-dependent mitochondrial degradation is substitutable in MEFs (see Discussion).

### p62/SQSTM1 is necessary for perinuclear clustering of damaged mitochondria

**Figure 7:**
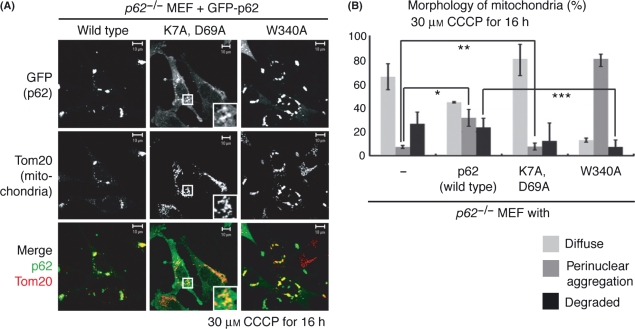
The PB1 domain of p62 is imperative for the clustering of depolarized mitochondria. (A) Immunocytochemistry of *p62*^*−/−*^ mouse embryonic fibroblasts (MEFs) complemented by wild-type p62 or the indicated p62 mutants. Higher magnification views of the boxed areas are shown in the insets in the middle panel. (B) The mitochondrial morphology of *p62*^*−/−*^ MEFs expressing the indicated p62 mutants was analyzed in more than 100 cells per mutation. Bars represent the mean ± SD values of at least three experiments. Asterisk shows significant difference from control (*P* < 0.01; Student’s *t*-test) whereas two asterisks show no significance (*P* = 0.43 > 0.01), meaning that wild-type p62 restored the mitochondrial clustering, whereas the PB1 domain mutant (K7A, D69A) did not. Triple asterisks show significant difference from wild-type control (*P* = 0.005 < 0.01), meaning that mitochondrial degradation was impaired in *p62*^*−/−*^ MEFs complemented with the p62 W340A mutant.

Although we did not observe a distinct defect in the degradation of damaged mitochondria in *p62* KO MEFs, we noticed that the perinuclear clustering of depolarized mitochondria was almost completely inhibited in *p62* deficient MEFs (Fig. 6A–C). To exclude the possible effect of factors other than disruption of *p62* in this phenotype, we examined whether wild-type p62 rescued the *p62*^*−/−*^ MEF phenotype. Re-introduction of GFP-tagged p62 (Ichimura *et al.* 2008) complemented the perinuclear clustering of depolarized mitochondria in *p62*^*−/−*^ MEFs (Fig. 7), confirming that the observed defects were caused by the loss of p62.

p62 possesses an N-terminal Phox and Bem1 (PB1) domain that mediates hetero- and homo-oligomerization of p62 (Lamark *et al.* 2003) and the LC3 recognition sequence (LRS)/LC3 interacting region (LIR) identified in murine (Ichimura *et al.* 2008) and human p62 (Pankiv *et al.* 2007), respectively, to promote association with autophagic machinery. To examine the role of these domains in mitochondrial clustering, we introduced a K7A/D69A double mutation in the PB1 domain or a W340A mutation in LRS/LIR, and examined whether these p62 mutants complemented the mitochondrial clustering in *p62*^*−/−*^ MEFs. When introduced into *p62*^*−/−*^ MEFs, the K7A/D69A mutant was unable to complement the dispersion of mitochondria, whereas the W340A mutant did (Fig. 7). These results suggest that oligomerization of p62 but not interaction with LC3 is essential for the mitochondrial clustering triggered by Parkin. Interestingly, a delay in mitochondrial degradation was observed in *p62*^*−/−*^ MEFs complemented with the p62 mutant W340A (Fig. 7B), suggesting that p62 is involved in Parkin-mediated mitophagy. The defect in *p62*^*−/−*^ MEFs, however, is concealed by other functionally redundant protein(s) (see Discussion).

## Discussion

### Function of p62 in the degradation of depolarized mitochondria

Growing lines of evidence indicate that p62, a selective substrate of autophagy, acts as a shuttle protein that transports ubiquitylated proteins to the autophagosome via interactions with both the ubiquitin and autophagic component LC3 (Kirkin *et al.* 2009; Komatsu & Ichimura 2010). Because p62 is selectively transported to the depolarized mitochondria upon Parkin-catalyzed ubiquitylation, we initially predicted that p62 links Parkin-mediated ubiquitylation and mitophagy. This idea is based on the preceding paper regarding p62-mediated pexophagy and xenophagy i.e., a selective autophagy for cytoplasm-invading bacteria and superfluous peroxisomes (Kim *et al.* 2008; Yoshikawa *et al.* 2009; Zheng *et al.* 2009). However, in our experimental settings of this study, we showed by immunocytochemistry and quantitative analysis of mitochondrial DNA that Parkin-dependent mitochondrial degradation was not hindered in *p62* KO (*p62*^*−/−*^) MEFs (Figs 6,7). These results, while admittedly not conclusive, seemingly suggest that p62 is not involved in Parkin-mediated mitophagy. We think a key idea to resolve this discrepancy is functional redundancy. Even when p62 is lost, several intracellular proteins such as NBR1, Alfy and BAG3 (Kirkin *et al.* 2009) might be able to link Parkin-catalyzed ubiquitylation to autophagic degradation, and if so the degradation-incompetent phenotype of *p62*^*−/−*^ MEFs might be concealed. Interestingly, we noticed that mitochondrial degradation was impaired in *p62*^*−/−*^ MEFs complemented with the p62 W340A mutant, which cannot interact with LC3 (Fig. 7B). The most plausible explanation for this result is that the p62 W340A mutant competes for certain common factor(s) with functionally redundant protein(s), and thus has a dominant effect, implying the involvement of p62 in Parkin-mediated mitophagy (note that complete disruption of *p62* cannot cause this type of titration and thus cannot confer the dominant effect).

During the preparation of our manuscript, Geisler *et al.* (2010) reported that p62 is required for Parkin-dependent mitophagy. They showed that knockdown of *p62* in HeLa cells dramatically inhibited the final clearance of damaged mitochondria; cells with uncleared mitochondria decreased from 85% to 20% with *p62* siRNA treatment. Although our results are not consistent with Geisler *et al.*, methodological differences (i.e. we used *p62* knockout MEFs whereas they used *p62* siRNA in HeLa cells) may account for the conflicting observations and further analysis will clarify the reason for this discrepancy.

### Function of depolarized mitochondria juxtanuclear clustering

Although we did not observe a clear defect in mitochondrial degradation in *p62* KO MEFs, we realized that perinuclear clustering of depolarized mitochondria was severely inhibited by *p62* deletion, showing that p62 is involved in this process. Depolarized mitochondria were ubiquitylated by Parkin, recognized by p62, transported via microtubules to aggregate within a juxtanuclear region and sometimes degraded. This series of events is reminiscent of aggresome formation, a general cellular response to reduce the toxicity of misfolded or unassembled proteins. In this process, misfolded protein(s) are ubiquitylated, recognized by p62/NBR1 to be segregated and concentrated into proteinacious inclusion bodies and/or recognized by HDAC6 to be transported along microtubles, organized into a perinuclear aggregation (an aggresome) and sometimes degraded by autophagy (Johnston *et al.* 1998; Garcia-Mata *et al.* 1999; Olzmann *et al.* 2007; Kirkin *et al.* 2009). The similarity between aggresome and mitochondrial clustering, although superficial at the moment, suggests that the two ubiquitin-mediated cytoprotective events have something in common.

Then, what is the physiologic significance of the microtubule transport and juxtanuclear clustering of depolarized mitochondria? Two scenarios are possible. In the first scenario, this event is involved in inclusion body formation. Many neurodegenerative diseases typically involve deposits of inclusion bodies containing abnormal aggregated proteins. Such inclusion bodies have been suggested to be pathogenic; however, growing lines of evidence indicate that they are not the main cause of toxicity but a consequence of a protective cellular response (Ross & Poirier 2005). In the case of Parkinson disease, a characteristic inclusion called the Lewy body (LB) is observed within neurons of the substntia nigra. Interestingly, electron microscopic analysis by Gai *et al.* (2000) showed that many mitochondria are concentrated in the early forms of Lewy bodies and Bedford *et al.* (2008) also demonstrated deposits of mitochondria in the early form of the Lewy body (pale body) in patients with Parkinson’s disease. These results suggest that mitochondria are a key component of the early phase in the biogenesis of the Lewy body and thus mitochondrial clustering might be involved in this process.

In the second scenario, transport and perinuclear clustering of depolarized mitochondria in cultured cells reflect withdrawal of axonal mitochondria in neurons. Generally, mitochondria must be positioned properly to serve the needs of the cell. Neuron cells are highly polarized cells, and thus the importance of mitochondrial positioning looms much larger than conventional cells. Indeed, mitochondria are delivered to areas of the axon where metabolic demand is high, such as synapses, active growth cones and branches (Hollenbeck & Saxton 2005). In axons, microtubules are oriented with their plus ends toward the periphery, and damaged mitochondria are subjected to retrograde transport along microtubules toward the cell body, which is the same direction of aggresome formation (MacAskill & Kittler 2010). We speculate that the retrograde transport along microtubules and the juxtanuclear clustering of depolarized mitochondria mediated by PINK1/Parkin in cultured cells reflect the aforementioned retrieval of damaged mitochondria in neuronal axons. If so, then PINK1/Parkin functions not only in degradation but also in retrieval and quarantine of damaged mitochondria.

## Experimental procedures

### Cell culture and transfection

HeLa cells were cultured at 37 °C with 5% CO_2_ in Dulbecco’s modified Eagle’s medium (DMEM) supplemented with glucose and l-glutamine (Sigma) containing penicillin/streptomycin and 10% fetal bovine serum (FBS; Gibco). To obtain transformants, cells were transiently transfected with various plasmids by Fugene 6 (Roche). MEFs were cultured at 32 °C with 5% CO_2_ in MEF media containing DMEM, 10% FBS, penicillin/streptomycin, β-mercaptoethanol (Sigma), 1× nonessential amino acids (Gibco) and 1 m Sodium Pyruvate (Gibco). To induce the expression of wild-type or mutants p62 in *p62*^*−/−*^ MEFs (Ichimura *et al.* 2008), cells were pre-treated with 250 ng/mL of doxycycline (Sigma). Various stable transformants of MEFs were established by infecting MEFs with recombinant retroviruses. HA-Parkin and GFP-Parkin were cloned into a pMXs-puro vector. Retrovirus packaging cells, PLAT-E were provided by Dr T. Kitamura from the University of Tokyo (Kitamura *et al.* 2003) were transfected with the aforementioned vectors and were cultured at 37 °C for 24 h. After changing the medium, cells were further incubated at 37 °C for 24 h and the viral supernatant was collected and used for infection. MEFs were plated onto 35-mm dishes at 24 h before infection, and the medium was replaced with the undiluted viral supernatant described earlier with 8 μg/mL polybrene (Sigma). Two days later, transformants were selected in medium containing 10 μg/mL puromycin.

### Electron microscopic analysis

COX electronmicrographs were carried out as reported previously (Nonaka *et al.* 1989; Nakada *et al.* 2001). Briefly, cultured cells were fixed in cooled 0.05 m phosphate buffer (pH 7.4) with 2% glutaraldehyde for <1 min. The fixed samples were rinsed with 0.05 m phosphate buffer (pH 7.4) at room temperature and stained for COX activity (Seligman *et al.* 1968). The stained section was washed in PBS and fixed in OsO_4_ for 45 min. After dehydration, the samples were embedded in an epoxy resin. Ultrathin sections that were not stained with uranyl acetate and lead nitrate were directly observed.

### Measurement of mitochondrial DNA contents

Total genomic and mitochondrial DNA were purified from wild-type, *Atg7* ^*−/−*^ or *p62*^*−/−*^ MEFs expressing GFP-Parkin using Gentra Puregene Kit (QIAGEN) according to recommended protocols, and total DNA was adjusted to a concentration of 0.1–1 ng/μL in TE buffer was used as a PCR template. Real-time quantitative PCR was carried out using QuantiTect SYBR Green PCR kit (QIAGEN) with a set of primers for a reference gene, GAPDH (5′-AAC GAC CCC TTC ATT GAC -3′ and 5′-TCC ACG ACA TAC TCA GCA C-3′), or for the target gene, mitochondrial 12s ribosomal RNA (5′-AAC TCA AAG GAC TTG GCG GTA CTT TAT ATC-3′ and 5′-GAT GGC GGT ATA TAG GCT GAA TTA GCA AGA G-3′). To prevent carry-over contamination, 0.5 unit of Uracil DNA Glycosylase (Invitrogen) was added during PCR, and samples were prepared in triplicate for each condition. PCR was carried out with a 7900HT Sequence Detection System (Applied Biosystems) under the following conditions: initial activation at 50 °C for 2 min and 95 °C for 15 min, amplification by 40 cycles of 95 °C for 20 s and 60 °C for 60 s. Data acquisition and analysis were carried out on a 7900HT SDS 2.0 (Applied Biosystems).

### Immunocytochemistry

To depolarize the mitochondria, HeLa cells were treated with 10 μm CCCP, and MEFs were treated with 30 μm CCCP for appropriate times. To depolymerize microtubules, HeLa cells were pre-treated with 50 μg/mL colchicine or 10 μg/mL nocodazole for 16 h and then were treated with 10 μm CCCP plus each inhibitor for 4 h. To check the specificity of linkage-specific anti-ubiquitin antibodies, Lys 48- or Lys 63-linked polyubiquitin chain (Enzo Life Sciences) was used as a positive control and subjected to immunoblotting.

For immunofluorescence experiments, cells were fixed with 4% paraformaldehyde, permeabilized with 50 μg/mL digitonin and stained with primary antibodies described in the next section and with the following secondary antibodies: mouse, rabbit and/or guinea pig Alexa Fluor 488, 568 and 647 (Invitrogen). The N-terminal 34 amino acids of PINK1 were fused to GFP to stain mitochondria in some triple staining experiments. Cells were imaged using a laser-scanning microscope (LSM510 META; Carl Zeiss, Inc.) with a Plan-Apochromat 63 × NA 1.4 oil differential interference contrast objective lens. Image contrast and brightness were adjusted in Photoshop (Adobe).

To inhibit the autophagic and lysosomal pathway, 0.1 μm bafilomycin A_1_ (Calbiochem) or a mixture of 10 μg/mL E64d, 10 μg/mL pepstatin A and 10 mm NH_4_Cl (Sigma) was added with CCCP.

### Antibodies

Antibodies used in this study are as follows: anti-GFP (3E6; Wako chemical), anti-HA [12CA5 (Roche); or F7 (Santa Cruz)], anti-Parkin (#2132; Cell Signaling), anti-Tom20 (FL-145 and F-10; Santa Cruz), anti-Ubiquitin [P4D1 (Santa Cruz) for immunoblotting; or FK2 (MBL) for immunocytochemistry], anti-K48-linked polyubiquitin (Apu2; Millipore), anti-K63-linked polyubiquitin [HWA4C4 (BIOMOL); or Apu3 (Millipore)] and anti-p62 (PROGEN).

## Author note

After our manuscript had been accepted, Lee *et al.* (2010) independently published results that are related to those described herein.

## Supporting Information/Supplementary material

The following Supporting Information can be found in the online version of the article:

**Figure S1** Merged data shown in Fig. 4 are not derived from channel crosstalk.

## References

[b1] Bedford L, Hay D, Devoy A (2008). Depletion of 26S proteasomes in mouse brain neurons causes neurodegeneration and Lewy-like inclusions resembling human pale bodies. J. Neurosci..

[b2] Bjorkoy G, Lamark T, Brech A, Outzen H, Perander M, Overvatn A, Stenmark H, Johansen T (2005). p62/SQSTM1 forms protein aggregates degraded by autophagy and has a protective effect on huntingtin-induced cell death. J. Cell Biol..

[b3] Clark IE, Dodson MW, Jiang C, Cao JH, Huh JR, Seol JH, Yoo SJ, Hay BA, Guo M (2006). Drosophila pink1 is required for mitochondrial function and interacts genetically with parkin. Nature.

[b4] Denison SR, Wang F, Becker NA, Schule B, Kock N, Phillips LA, Klein C, Smith DI (2003). Alterations in the common fragile site gene Parkin in ovarian and other cancers. Oncogene.

[b5] Gai WP, Yuan HX, Li XQ, Power JT, Blumbergs PC, Jensen PH (2000). *In situ* and *in vitro* study of colocalization and segregation of alpha-synuclein, ubiquitin, and lipids in Lewy bodies. Exp. Neurol..

[b6] Garcia-Mata R, Bebok Z, Sorscher EJ, Sztul ES (1999). Characterization and dynamics of aggresome formation by a cytosolic GFP-chimera. J. Cell Biol..

[b7] Geisler S, Holmstrom KM, Skujat D, Fiesel FC, Rothfuss OC, Kahle PJ, Springer W (2010). PINK1/Parkin-mediated mitophagy is dependent on VDAC1 and p62/SQSTM1. Nat. Cell Biol..

[b8] Hampe C, Ardila-Osorio H, Fournier M, Brice A, Corti O (2006). Biochemical analysis of Parkinson’s disease-causing variants of Parkin, an E3 ubiquitin-protein ligase with monoubiquitylation capacity. Hum. Mol. Genet..

[b9] Hirokawa N (1998). Kinesin and dynein superfamily proteins and the mechanism of organelle transport. Science.

[b10] Hollenbeck PJ, Saxton WM (2005). The axonal transport of mitochondria. J. Cell Sci..

[b11] Ichimura Y, Kumanomidou T, Sou YS, Mizushima T, Ezaki J, Ueno T, Kominami E, Yamane T, Tanaka K, Komatsu M (2008). Structural basis for sorting mechanism of p62 in selective autophagy. J. Biol. Chem..

[b12] Johnston JA, Ward CL, Kopito RR (1998). Aggresomes: a cellular response to misfolded proteins. J. Cell Biol..

[b13] Kim PK, Hailey DW, Mullen RT, Lippincott-Schwartz J (2008). Ubiquitin signals autophagic degradation of cytosolic proteins and peroxisomes. Proc. Natl Acad. Sci. USA.

[b14] Kirkin V, McEwan DG, Novak I, Dikic I (2009). A role for ubiquitin in selective autophagy. Mol. Cell.

[b15] Kitada T, Asakawa S, Hattori N, Matsumine H, Yamamura Y, Minoshima S, Yokochi M, Mizuno Y, Shimizu N (1998). Mutations in the parkin gene cause autosomal recessive juvenile parkinsonism. Nature.

[b16] Kitamura T, Koshino Y, Shibata F, Oki T, Nakajima H, Nosaka T, Kumagai H (2003). Retrovirus-mediated gene transfer and expression cloning: powerful tools in functional genomics. Exp. Hematol..

[b17] Komatsu M, Ichimura Y (2010). Physiological significance of selective degradation of p62 by autophagy. FEBS Lett..

[b18] Komatsu M, Waguri S, Koike M (2007). Homeostatic levels of p62 control cytoplasmic inclusion body formation in autophagy-deficient mice. Cell.

[b19] Komatsu M, Waguri S, Ueno T, Iwata J, Murata S, Tanida I, Ezaki J, Mizushima N, Ohsumi Y, Uchiyama Y, Kominami E, Tanaka K, Chiba T (2005). Impairment of starvation-induced and constitutive autophagy in Atg7-deficient mice. J. Cell Biol..

[b20] Lamark T, Perander M, Outzen H, Kristiansen K, Overvatn A, Michaelsen E, Bjorkoy G, Johansen T (2003). Interaction codes within the family of mammalian Phox and Bem1p domain-containing proteins. J. Biol. Chem..

[b21] Lee JY, Nagano Y, Taylor JP, Lim KL, Yao TP (2010). Disease-causing mutations in parkin impair mitochondrial ubiquitination, aggregation, and HDAC6-dependent mitophagy. J. Cell Biol..

[b22] Lim KL (2007). Ubiquitin-proteasome system dysfunction in Parkinson’s disease: current evidence and controversies. Expert Rev. Proteomics.

[b23] MacAskill AF, Kittler JT (2010). Control of mitochondrial transport and localization in neurons. Trends Cell Biol..

[b24] Matsuda N, Kitami T, Suzuki T, Mizuno Y, Hattori N, Tanaka K (2006). Diverse effects of pathogenic mutations of Parkin that catalyze multiple monoubiquitylation *in vitro*. J. Biol. Chem..

[b25] Matsuda N, Sato S, Shiba K, Okatsu K, Saisho K, Gautier CA, Sou Y-S, Saiki S, Kawajiri S, Sato F, Kimura M, Komatsu M, Hattori N, Tanaka K (2010). PINK1 stabilized by mitochondrial depolarization recruits Parkin to damaged mitochondria and activates latent Parkin for mitophagy. J. Cell Biol..

[b26] Matsuda N, Tanaka K (2010). Does impairment of the ubiquitin-proteasome system or the autophagy-lysosome pathway predispose individuals to neurodegenerative disorders such as Parkinson’s disease?. J. Alzheimers Dis..

[b27] Mizushima N, Yoshimori T, Levine B (2010). Methods in mammalian autophagy research. Cell.

[b28] Nakada K, Inoue K, Ono T, Isobe K, Ogura A, Goto YI, Nonaka I, Hayashi JI (2001). Inter-mitochondrial complementation: mitochondria-specific system preventing mice from expression of disease phenotypes by mutant mtDNA. Nat. Med..

[b29] Narendra D, Tanaka A, Suen DF, Youle RJ (2008). Parkin is recruited selectively to impaired mitochondria and promotes their autophagy. J. Cell Biol..

[b30] Narendra DP, Jin SM, Tanaka A, Suen DF, Gautier CA, Shen J, Cookson MR, Youle RJ (2010). PINK1 Is selectively stabilized on impaired mitochondria to activate parkin. PLoS Biol..

[b31] Newton K, Matsumoto ML, Wertz IE (2008). Ubiquitin chain editing revealed by polyubiquitin linkage-specific antibodies. Cell.

[b32] Noda NN, Kumeta H, Nakatogawa H, Satoo K, Adachi W, Ishii J, Fujioka Y, Ohsumi Y, Inagaki F (2008). Structural basis of target recognition by Atg8/LC3 during selective autophagy. Genes Cells.

[b33] Nonaka I, Koga Y, Ohtaki E, Yamamoto M (1989). Tissue specificity in cytochrome c oxidase deficient myopathy. J. Neurol. Sci..

[b34] Olzmann JA, Li L, Chudaev MV, Chen J, Perez FA, Palmiter RD, Chin LS (2007). Parkin-mediated K63-linked polyubiquitination targets misfolded DJ-1 to aggresomes via binding to HDAC6. J. Cell Biol..

[b35] Pankiv S, Clausen TH, Lamark T, Brech A, Bruun JA, Outzen H, Overvatn A, Bjorkoy G, Johansen T (2007). p62/SQSTM1 binds directly to Atg8/LC3 to facilitate degradation of ubiquitinated protein aggregates by autophagy. J. Biol. Chem..

[b36] Park J, Lee SB, Lee S, Kim Y, Song S, Kim S, Bae E, Kim J, Shong M, Kim JM, Chung J (2006). Mitochondrial dysfunction in Drosophila PINK1 mutants is complemented by parkin. Nature.

[b37] Pickart C, Raasi S (2005). Controlled synthesis of polyubiquitin chains. Methods Enzymol..

[b38] Ross CA, Poirier MA (2005). Opinion: what is the role of protein aggregation in neurodegeneration?. Nat. Rev. Mol. Cell Biol..

[b39] Saeki Y, Kudo T, Sone T, Kikuchi Y, Yokosawa H, Toh-e A, Tanaka K (2009). Lysine 63-linked polyubiquitin chain may serve as a targeting signal for the 26S proteasome. EMBO J..

[b40] Seligman AM, Karnovsky MJ, Wasserkrug HL, Hanker JS (1968). Nondroplet ultrastructural demonstration of cytochrome oxidase activity with a polymerizing osmiophilic reagent, diaminobenzidine (DAB). J. Cell Biol..

[b41] Shimura H, Hattori N, Kubo S, Mizuno Y, Asakawa S, Minoshima S, Shimizu N, Iwai K, Chiba T, Tanaka K, Suzuki T (2000). Familial Parkinson disease gene product, parkin, is a ubiquitin-protein ligase. Nat. Genet..

[b42] Shvets E, Fass E, Scherz-Shouval R, Elazar Z (2008). The N-terminus and Phe52 residue of LC3 recruit p62/SQSTM1 into autophagosomes. J. Cell Sci..

[b43] Twig G, Elorza A, Molina AJ (2008). Fission and selective fusion govern mitochondrial segregation and elimination by autophagy. EMBO J..

[b44] Valente EM, Abou-Sleiman PM, Caputo V (2004). Hereditary early-onset Parkinson’s disease caused by mutations in PINK1. Science.

[b45] Vives-Bauza C, Zhou C, Huang Y (2010). PINK1-dependent recruitment of Parkin to mitochondria in mitophagy. Proc. Natl Acad. Sci. USA.

[b46] Wang H, Matsuzawa A, Brown SA, Zhou J, Guy CS, Tseng PH, Forbes K, Nicholson TP, Sheppard PW, Hacker H, Karin M, Vignali DA (2008). Analysis of nondegradative protein ubiquitylation with a monoclonal antibody specific for lysine-63-linked polyubiquitin. Proc. Natl Acad. Sci. USA.

[b47] Weissman AM (2001). Themes and variations on ubiquitylation. Nat. Rev. Mol. Cell Biol..

[b48] Yang Y, Gehrke S, Imai Y, Huang Z, Ouyang Y, Wang JW, Yang L, Beal MF, Vogel H, Lu B (2006). Mitochondrial pathology and muscle and dopaminergic neuron degeneration caused by inactivation of Drosophila Pink1 is rescued by Parkin. Proc. Natl Acad. Sci. USA.

[b49] Yoshikawa Y, Ogawa M, Hain T, Yoshida M, Fukumatsu M, Kim M, Mimuro H, Nakagawa I, Yanagawa T, Ishii T, Kakizuka A, Sztul E, Chakraborty T, Sasakawa C (2009). Listeria monocytogenes ActA-mediated escape from autophagic recognition. Nat. Cell Biol..

[b50] Zheng YT, Shahnazari S, Brech A, Lamark T, Johansen T, Brumell JH (2009). The adaptor protein p62/SQSTM1 targets invading bacteria to the autophagy pathway. J. Immunol..

[b51] Ziviani E, Tao RN, Whitworth AJ (2010). Drosophila Parkin requires PINK1 for mitochondrial translocation and ubiquitinates Mitofusin. Proc. Natl Acad. Sci. USA.

