# Supplementary figures and images for: p62/SQSTM1 cooperates with Parkin for perinuclear clustering of depolarized mitochondria
Source: Genes Cells. 2010 Aug;15(8):887–900. doi: 10.1111/j.1365-2443.2010.01426.x (PMC2970908; doi:10.1111/j.1365-2443.2010.01426.x)

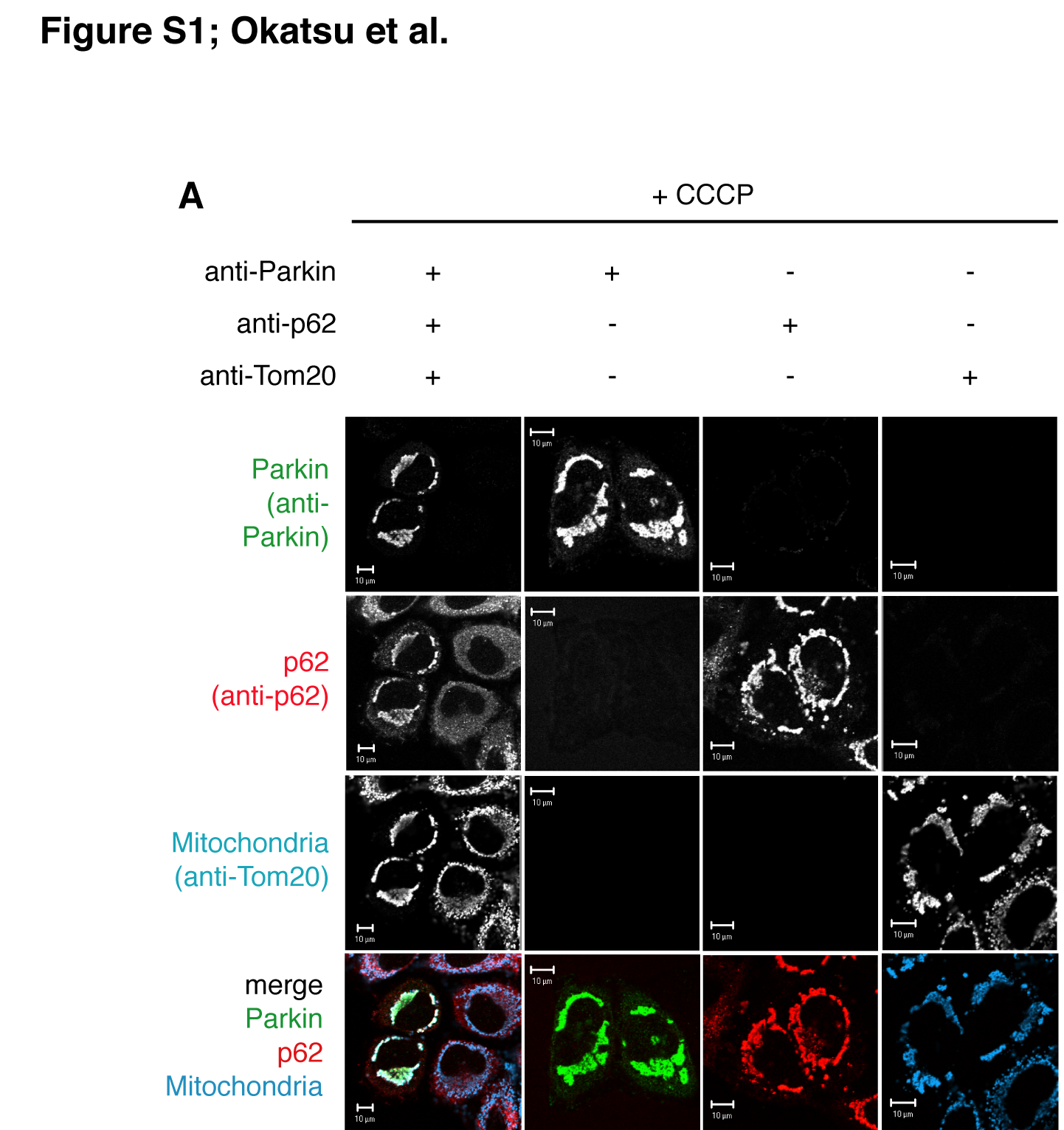

Supplement: Supplementary file 1 [file gtc0015-0887-SD1.tif]
